# Psychological affection in rheumatoid arthritis patients in relation to disease activity: Erratum

**DOI:** 10.1097/MD.0000000000016515

**Published:** 2019-07-12

In the article, “Psychological affection in rheumatoid arthritis patients in relation to disease activity”,^[1]^ which appears in Volume 98, Issue 19 of *Medicine*, Dr. Ahmed Mohmed Kamal's name appeared incorrectly as Ahmed Mustafa Kamal.
